# Snail Mucus-Inspired Interface: A Resilient and Self-Healing Double-Network Hydrogel Polymer Electrolyte for Flexible Supercapacitors

**DOI:** 10.3390/gels12050441

**Published:** 2026-05-17

**Authors:** Mengxiao Wang, Jia Yang, Gang Qin, Qiang Chen

**Affiliations:** 1 School of Electrical Engineering, Zhejiang University of Water Resources and Electric Power, Hangzhou 310018, China; wangmx@zuwe.edu.cn; 2 School of Materials Science and Engineering, Henan Polytechnic University, Jiaozuo 454000, China; jiangshanjia@hpu.edu.cn; 3 Wenzhou Institute, University of Chinese Academy of Sciences, Wenzhou 352001, China

**Keywords:** double-network hydrogel, hydrogel polymer electrolyte, self-healing, snail mucus-inspired interface, flexible supercapacitor

## Abstract

Flexible supercapacitors (SCs) have attracted considerable attention for wearable electronics, and developing high-performance electrolytes is critical for their practical application. While hydrogels have been widely investigated as solid electrolytes, studies on double-network (DN) hydrogel electrolytes specifically addressing the electrode–electrolyte interface stability under mechanical deformation remain relatively scarce. A major obstacle is maintaining a stable electrode–electrolyte interface under large mechanical deformation. Drawing inspiration from the mucus of a snail, which effectively adheres to various surfaces in challenging conditions, we present a self-healing xanthan gum/hydrophobically associated polyacrylamide/NaCl (XG/HPAAm/NaCl) hydrogel polymer electrolyte (HPE) that facilitates the creation of flexible SCs with improved mechanical and electrochemical properties. The optimized 2 wt% XG/HPAAm/0.4 M NaCl DN HPE exhibits a high ionic conductivity of 4.0 S/m, a tensile strength of 0.43 MPa, and an elongation at break of 11.7 mm/mm, along with a high adhesive energy of 254.7 J/m^2^. The tough HPE was coated with a mixed adhesive of 502 cyanoacrylate glue and triethyl citrate (TEC) to create a surface coating resembling “mucus”, onto which activated carbon (AC)-modified carbon cloth (CC) electrodes (CC/AC) were affixed on both sides to construct the flexible SCs. Investigations into the HPE’s characteristics and the SCs’ electrochemical performance at various bending angles reveal that the “mucus-coating” HPE exhibits strong electrode adhesion and significantly improved electrochemical performance. The assembled flexible SC delivers a high specific capacitance of 249.3 F/g at 0.30 A/g, retains 73.4% of its initial capacitance after 20,000 cycles, and maintains 86.9% capacitance retention under 180° bending, outperforming SCs assembled with original HPEs in both performance and stability. This approach provides a versatile method for improving the interfacial properties between electrodes and HPEs, paving the way for innovative applications in robust, self-healing, and flexible devices.

## 1. Introduction

Hydrogels consist of three-dimensional (3D) networks of interconnected molecules, primarily made from polymers. These networks are established through either covalent bonds or non-covalent interactions [1,2]. The essential physicochemical properties of hydrogels, including mechanical strength, toughness, and stretchability, can be well tuned, and their high ionic conductivity and good interfacial contact make them promising electrolytes for electrochemical energy storage [3,4,5]. Recently, double-network (DN) tough hydrogels have attracted significant interest as promising candidates for wearable technology due to their excellent mechanical properties and functionalities [6,7]. These hydrogels, formed from two polymer networks that are either physically or chemically linked, demonstrate improved tensile strength, high energy absorption upon fracture, and enhanced toughness, while achieving a high stretchability of up to 2000% strain, far exceeding the limitations of conventional single-network hydrogels. Additionally, the natural porosity of hydrogels allows them to retain substantial volumes of water, which enhances their ionic conductivity, biocompatibility, and efficient mass transport. Consequently, tough hydrogels find extensive applications in flexible devices such as strain sensors [8,9], epidermal patch electrodes [10], triboelectric nanogenerators [11], energy storage systems [12,13], and gas sensors [14].

The increasing need for integrated energy storage solutions has become critical for enabling fully autonomous wearable technologies. Supercapacitors (SCs), in particular, are ideal as they offer rapid charging, safety, and convenience for wearable devices. Key to the development of flexible all-solid-state devices are solid-state gel-like polymers that incorporate aqueous electrolyte [15,16]. To enhance flexibility, it is essential to design electrode layers that are either bendable or stretchable, while tough hydrogel electrolytes are gaining attention for their role in flexible SCs [17,18]. For instance, Lin et al. [19] developed lithium-infused agarose/polyacrylamide (Li-AG/PAAm) DN hydrogel electrolytes through a combination of heating-cooling processes and radiation-induced polymerization. These electrolytes demonstrated favorable mechanical properties, including a compressive strength of 150 MPa, a break strain exceeding 99.9%, a tensile strength of 1103 kPa, and an elongation at break of 2780%, along with a high ionic conductivity of 41 mS·cm^−1^. SCs utilizing Li-AG/PAAm DN hydrogel electrolytes paired with activated carbon-coated nickel foam electrodes maintained stable electrochemical performance across various compressive strains and bending angles, even after enduring 1000 cycles of compression and bending. Similarly, Lu et al. [20] created a DN hydrogel featuring nanoparticle-cross-linked HPAAm as the primary network and Zn^2+^-cross-linked alginate as the secondary network. By soaking this hydrogel in a 3 M ZnSO_4_ solution, they produced a flexible electrolyte (with a tensile strain of 1572%) that exhibited moderate conductivity. SCs made with this electrolyte and carbon cloth electrodes showed stable capacitance retention during tensile, compressive, and bending stresses, achieving over 87% retention after 4000 charge–discharge cycles. Zhou et al. [21] prepared a DN hydrogel electrolyte by dissolving polyvinyl alcohol (PVA) and PAAm in a 2 M zinc chloride solution, incorporating xanthan gum (XG) with a distinctive trisaccharide side chain into the semi-interpenetrating network. This electrolyte achieved an ionic conductivity of 30.98 mS·cm^−1^, with a tensile fracture strength of up to 1.14 MPa and a fracture strain of 603%. When integrated into an SC with activated carbon-coated titanium foil as the cathode and zinc foil as the anode, the specific capacitance experienced only a minor reduction when tested under various bending angles (0° to 180°). Additionally, DN tough hydrogels have also introduced innovative features for energy storage, such as self-healing and cold-resistant SCs, as well as secure energy storage solutions [22,23,24]. While significant research has focused on flexible supercapacitors utilizing PAAm hydrogel electrolytes, there is a notable scarcity of studies on flexible SCs employing tough HPAAm-based hydrogel electrolytes with hydrophobic cross-linking in the existing literature.

A significant obstacle in utilizing tough hydrogels for flexible devices is achieving effective interfacial adhesion between the hydrogels and different materials, which prior research has addressed through the use of chemical adhesive layers [25,26]. In this study, we developed a durable DN hydrogel polymer electrolyte (HPE) composed of XG/HPAAm/NaCl, inspired by the mucus secretion mechanism of snails, and proposed a “mucus-coating” physical interaction strategy for advancing flexible SCs. This hydrogel serves a dual function as both a solid polymer electrolyte and a flexible structural support. By examining the electrochemical performance of the device at various bending angles, we illustrated that the integration of tough hydrogels with surface “mucus-coating” can enhance the mechanical, electrochemical properties, and cyclic stability of flexible SCs. Furthermore, we demonstrated the potential of this hydrogel-based power source to power light-emitting diodes (LEDs) through a proof-of-concept experiment. This work presents two complementary levels of innovation: (1) At the material level, the DN hydrogel electrolyte provides high mechanical toughness, stretchability, and ionic conductivity as a robust flexible substrate. (2) At the interface level, the TEC-modified 502 “mucus-coating” strategy—the core source of performance enhancement—addresses the long-standing problem of electrode–electrolyte delamination under deformation. Unlike conventional chemical adhesives that form dense, insulating films, this strategy achieves strong adhesion while preserving ion transport pathways, offering a simple yet effective solution for high-stability flexible supercapacitors.

## 2. Results and Discussion

### 2.1. Design Principle

**Figure 1:**
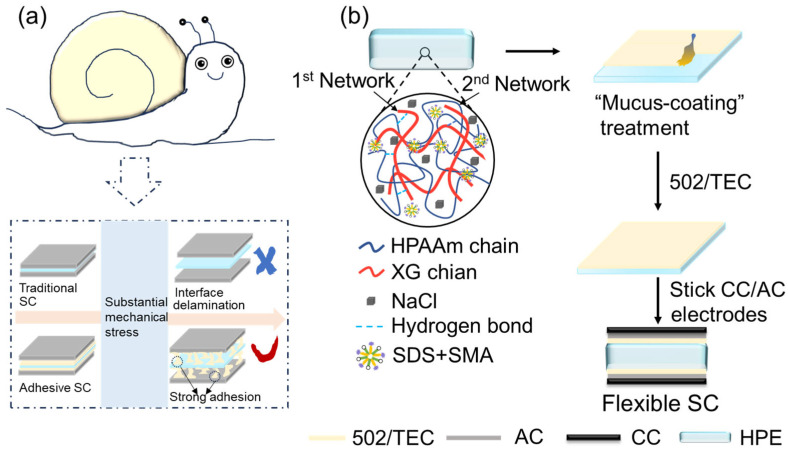
(**a**) A conceptual representation of a design for flexible SCs inspired by the mucus of a snail, utilizing tough hydrogel electrolytes featuring stable adhesive surfaces. (**b**) A diagram depicting the XG/HPAAm/NaCl tough HPE used in the creation of flexible, stretchable SCs through a method involving “mucus-coating”.

Drawing inspiration from the design of snail mucus that facilitates stable attachment to surfaces, we propose that tough hydrogels featuring a resemblance to “mucus-coating” could serve as a versatile solution to enhance the interfacial characteristics between electrodes and electrolytes, thereby improving the mechanical stability of flexible SCs (Figure 1a). The HPE underwent a “mucus” treatment using a direct coating technique to form an adhesive surface, followed by the attachment of AC-modified CC electrodes (CC/AC) on either side to create a flexible, stretchable SC device (Figure 1b). Notably, this mucus-inspired coating is the key interfacial innovation of this work. Unlike traditional adhesives that form dense, insulating films, the TEC-modified 502 glue cures into a thin, uniform interfacial layer that provides strong adhesion while preserving ion transport pathways. This strategy is simple, scalable, and avoids the need for complex surface modification, making it highly suitable for flexible energy storage devices.

### 2.2. Ionic Conductivity and Mechanical Properties of the XG/HPAAm/NaCl DN HPE

**Figure 2:**
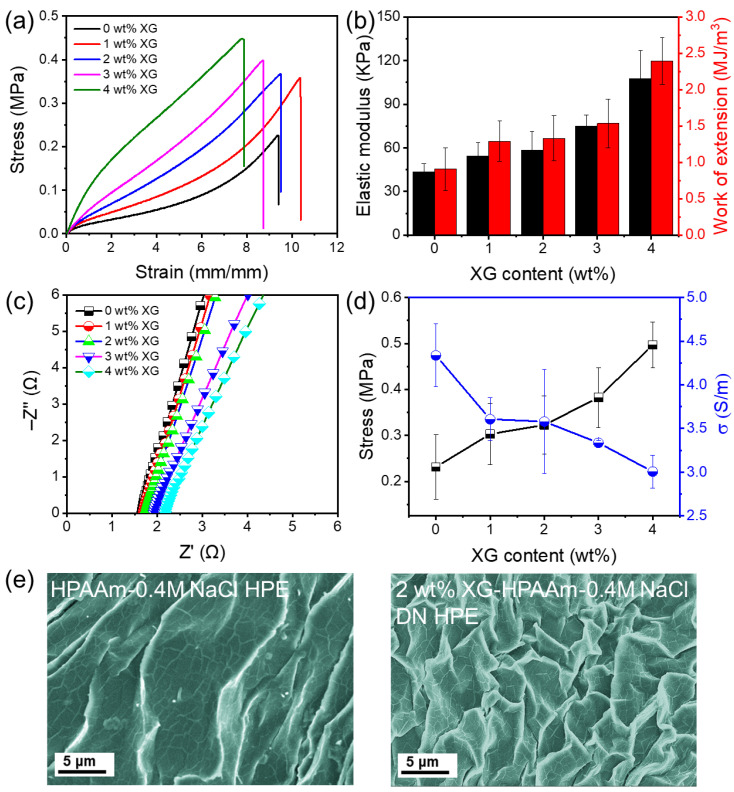
Effect of XG concentration on (**a**) the tensile stress–strain curves, (**b**) the elastic modulus and work of extension, (**c**) EIS curves, (**d**) stress and ionic conductivity of XG/HPAAm/NaCl DN HPE membranes (0.4 M NaCl). (**e**) SEM photographs of the HPAAm-0.4M NaCl HPE and the 2 wt% XG/HPAAm/0.4M NaCl DN HPE.

Figure 2a illustrates the strain–stress characteristics of XG/HPAAm/NaCl DN HPEs with varying XG concentrations from 0 to 4 wt%. The presence of micelles rendered the HPAAm hydrogel without XG soft, exhibiting an elongation at break of 9.4 mm/mm and a fracture stress of 0.23 MPa, which is significantly higher than that of conventional chemically crosslinked PAAm hydrogels [27,28]. In contrast, the 1 wt% XG/PAAm/NaCl DN HPE demonstrated a fracture stress of 0.36 MPa, representing a 1.6-fold increase compared to the pure HPAAm single network HPE. The elongation at break for the 1 wt% XG/HPAAm/NaCl was recorded at 10.4 mm/mm, marginally exceeding that of the pure HPAAm hydrogel. The mechanical properties of the hydrogels were significantly influenced by the XG concentration. As the XG content increased, both the elastic modulus and work of extension improved (Figure 2b), suggesting that the initial network of XG chains significantly enhanced mechanical strength. This improvement in mechanical properties is likely due to the more cohesive XG-HPAAm DN network, which effectively disperses and transfers stress, leading to enhanced mechanical reinforcement [29].

Figure 2c presents the electrochemical impedance spectroscopy (EIS) curves for the XG/HPAAm/NaCl DN HPE with varying amounts of XG. It is evident that as the XG concentration rises, the resistance (*R*_b_) of the HPE also increases, while conductivity shows a decline, as depicted in Figure 2d. The detailed numerical data are summarized in Table S1. The higher XG levels contribute to a denser porous network in the HPE, resulting in reduced water content, which negatively impacts ion transport [30]. SEM images of the PAAm and 2 wt% XG/HPAAm/NaCl HPEs are illustrated in Figure 2e. In the HPAAm/0.4 M NaCl HPE, the pore walls appear relatively thin. Conversely, the 2 wt% XG/HPAAm/0.4 M NaCl HPE displays distinct morphologies due to the establishment of a DN. The incorporation of XG significantly reduced the pore size and led to a more uniform pore distribution, indicating a more compact and consistent structure. Additionally, electrochemical stability is a crucial factor. As shown in Figure S1, there is minimal faradaic current in the cyclic voltammetry (CV) curves within the −2 to +2 V range, suggesting that the stability window for the 2 wt% XG/HPAAm/NaCl DN HPE is approximately 2.4 V and is not significantly influenced by the amount of XG present.

A comprehensive investigation was conducted on the mechanical characteristics of a 2 wt% XG/HPAAm/NaCl DN HPE membrane with varying NaCl concentrations to identify the ideal ratio. As illustrated in Figure S2a,b, the tensile strength initially rises and then falls, peaking at 0.43 MPa when the NaCl concentration is 0.4 M, surpassing the 0.31 MPa of the 2 wt% XG/HPAAm membrane. Additionally, the elongation at break reaches 11.7 mm/mm, indicating that incorporating NaCl contributes to the improved mechanical properties of the HPE. This enhancement occurs because the XG molecular chains possess negative charges (such as carboxylate groups), which are neutralized by Na^+^ ions, thereby diminishing the electrostatic repulsion among the chains and allowing them to come together more closely [31]. This charge neutralization strengthens intermolecular interactions and results in a more robust network structure, thus increasing tensile strength [32].

However, the strength decreases when the NaCl concentration exceeds 0.4 M, and a decline in strength is observed. This reduction is attributed to the potential loss of uniform dispersion of XG molecules at high salt levels, leading to excessive aggregation and a discontinuous gel structure, which ultimately compromises the material’s overall tensile properties [32,33]. Furthermore, as illustrated in Figure S2c, the 2 wt% XG/HPAAm/0.4 M NaCl DN HPE membrane exhibits good mechanical characteristics, capable of enduring deformations such as stretching, twisting, and knotting without any visible damage. This highlights the hydrogel’s good softness and toughness. The influence of NaCl concentration on the EIS curves and the ionic conductivity is presented in Figure S3a,b. The resistance of the HPE decreased as the NaCl concentration increased from 0.0 to 0.8 M, while the ionic conductivity increased from 1.9 to 4.0 S/m. Corresponding data are listed in Table S2. This enhancement is attributed to the increased availability of mobile ions (Na^+^ and Cl^−^) within the hydrogel due to the addition of NaCl, which boosts conductivity [34]. Additionally, assessing the electrochemical stability of HPEs is crucial for their practical use. As depicted in Figure S4, there is an absence of significant Faraday current appearing in the range of approximately −1.2 to +1.2 V, suggesting that the stable voltage window of the HPE is around 2.4 V and remains largely unaffected by the concentration of NaCl.

### 2.3. Self-Healability of the XG/HPAAm/NaCl DN HPE

Due to the potential damage to HPEs and the subsequent failure of energy-storage devices caused by external factors like bending, stretching, and cutting, it is essential to create HPEs that possess self-healing properties [35]. A DN HPE composed of 2 wt% XG/HPAAm/0.4 M NaCl was sliced into two pieces. These pieces were then aligned without any pressure and allowed to rest at various temperatures for specified durations. The tensile testing was performed on the repaired HPE samples to assess their self-healing capabilities.

As illustrated in Figures S5a and S6a, the duration for repair time was one hour. The restored 2 wt% XG/HPAAm/0.4 M NaCl DN HPE exhibits stress values of 0.02 and 0.08 MPa, along with strains of 0.5 and 2.8 mm/mm at temperatures of 30 and 50 °C, respectively. The healing efficiency for elongation is recorded at 5% and 15%, as shown in Figure S6b. The second network is engineered to form through reversible, noncovalent bonds, which arise from strong hydrophobic interactions between SDS micelles and the alkyl groups of SMA side chains. These temporarily dissociated noncovalent bonds in the second HPAAm network can quickly reform at ambient temperature without requiring external stimuli [29]. The increase in healing efficiency with temperature is due to the enhanced mutual diffusion of polymer chains at elevated temperatures, allowing them to intertwine across the interface of the two damaged HPE samples, thereby creating robust interactions [36]. These results demonstrate that the DN HPE exhibits moderate mechanical self-healing efficiency within 1 h. The conductivity of the 2 wt% XG/HPAAm/0.4 M NaCl DN HPE during the healing process is depicted in Figure S5b, showing a recovery of 87.6% of its initial ionic conductivity after 10 cutting-healing cycles. This indicates high ionic conductivity recovery, which helps maintain stable electrochemical performance even after minor damage, thus improving the durability of flexible supercapacitors. The effect of healing duration (see Figures S6c and S7a,b) on the self-repairing properties of the 2 wt% XG/HPAAm/0.4 M NaCl DN HPE is investigated. It is observed that both the tensile stress and strain of the repaired HPE rise as the healing time extends, attributed to the enhanced solubility of the hydrophobic components within the hydrogels and the increased mobility of the polymer chains at elevated temperatures over longer periods [30,37]. Consequently, the self-healing mechanism is illustrated in Figure S7c.

### 2.4. Electrochemical Performance of the XG/HPAAm/NaCl DN HPE-Based SC

**Figure 3:**
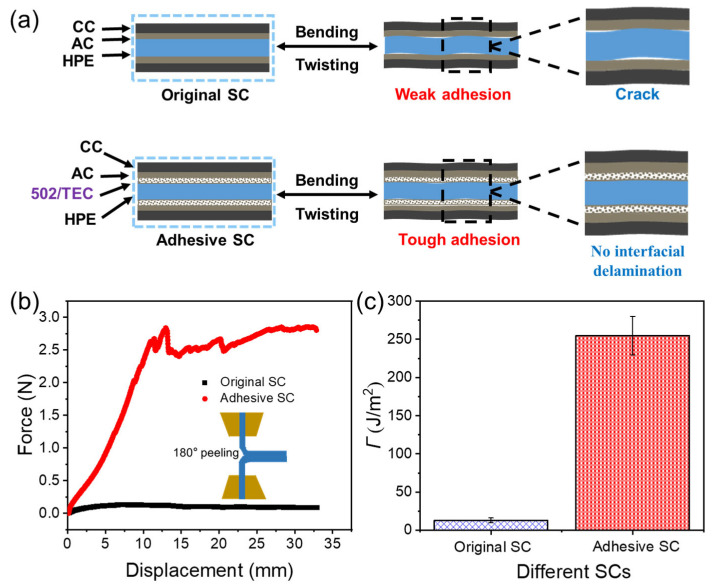
(**a**) Schematic diagram of the effect of interfacial adhesion between the electrode and HPE of original and adhesive SCs. Curves of the (**b**) peeling force and (**c**) adhesion energy for original and adhesive SCs.

Due to inadequate conformability and weak adhesion, interfacial voids and delamination occur between the HPE and the flexible electrode, leading to significant performance degradation of SCs under repeated deformation [38]. As illustrated in Figure 3a, we propose a strategy of applying an adhesive to the surface of the pre-stretched HPE to create a “mucus-coating” surface structure. This structure enhances the interfacial adhesion between the electrode and the electrolyte, thereby achieving long cycling durability of SCs. Figure 3b presents the relationship between force and displacement for two types of SCs. The original SC consistently exhibits a low force across all displacement levels, reaching approximately 0.087 N at the maximum displacement of 33 mm. In contrast, the adhesive SC demonstrates a pronounced mechanical response, peaking at around 2.83 N at a displacement of approximately 13 mm, and subsequently maintaining a relatively high force of about 2.65 N with only minor fluctuations. The adhesive energy (*Γ*) is determined by *Γ* = 2F/w, where F corresponds to the steady-state peeling force, and w denotes the width of the sample. As shown in Figure 3c, the Γ value is close to 0 for the original SC, which means there is almost no effective bonding between the electrode and electrolyte. In contrast, the adhesive SC displays a significant enhancement in Γ, reaching ~254.7 J/m^2^, indicating that the interface modification effectively enhances the interfacial toughness between the electrode and electrolyte. Such a robust interface bonding can avoid interface separation or voids under deformation, which is critical for maintaining the electrochemical performance and structural stability of SCs.

The electrochemical characteristics of the XG/HPAAm/NaCl DN HPE SC were evaluated using techniques such as EIS, CV, Galvanostatic charge–discharge (GCD), and tests for cycling stability. The EIS data (Figure S8a) reveal linear patterns at low frequencies and semicircular shapes at high frequencies. In comparison to the original SC (*R*s, 9.10 Ω), the adhesive SC variant demonstrates a reduced internal resistance (*R*_s_, 8.31 Ω). Furthermore, it exhibits a lower charge transfer resistance (*R*_ct_, 1.45 Ω), as evidenced by the semicircle’s width along the *x*-axis. Additionally, the slopes observed at mid- and low frequencies provide insights into ion diffusion resistance and the capacitive behavior. The adhesive SC sample displayed steeper slopes than the original, indicating enhanced ionic diffusion and performance that aligns more closely with that of an ideal electric double layer capacitor (EDLC) [39].

**Figure 4:**
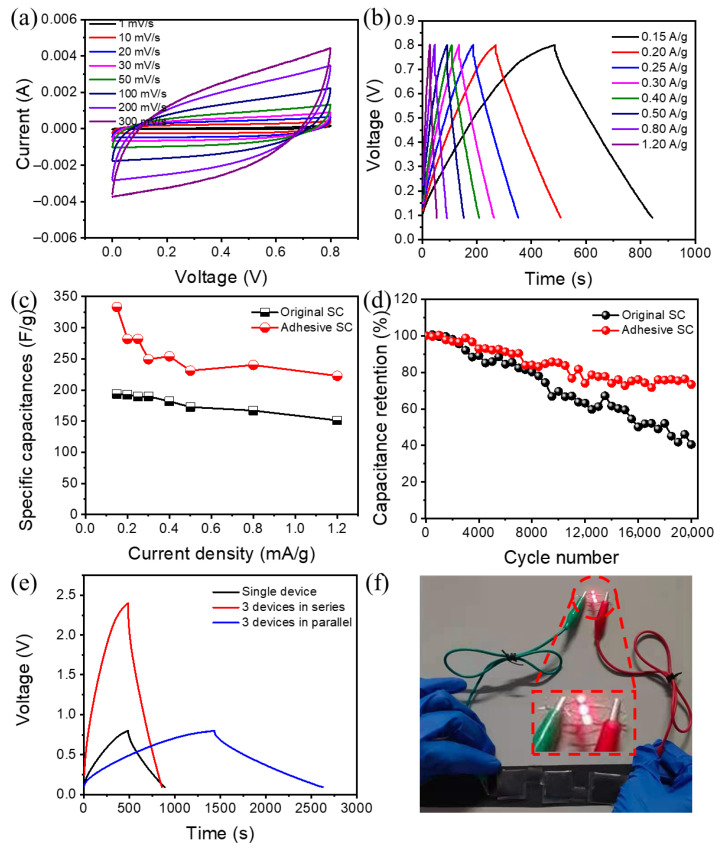
Electrochemical characteristics of original and adhesive SCs. (**a**) CV curves of the adhesive SC at various scan rates from 1 to 300 mV/s; (**b**) GCD curves of the adhesive SC at various current densities from 0.15 to 1.2 A/g; (**c**) specific capacitance values at varying current densities at a voltage of 0.8 V; (**d**) cycling performance at 0.50 A/g. (**e**) GCD curves of a single adhesive SC and a group of three adhesive SCs in series and parallel at the current density of 0.15 A/g; (**f**) Photograph of an LED lit up by the three adhesive SCs in series.

Figure S8b and Figure 4a present the CV curves for both types of SCs across different scan rates. The CV curves for the adhesive SC exhibit a nearly rectangular and balanced form, which suggests a low *R*_ct_ and characteristic EDLC behavior. Notably, the total area under the CV curve for the adhesive SC is significantly greater than that of the original SC, indicating enhanced specific capacitance attributed to the “mucus-coating” design. As the scan rates increase, the CV profiles progressively lose their rectangular shape, a phenomenon linked to ionic transport being diffusion-controlled [40]. The GCD curves (see Figure S8c and Figure 4b) reveal that the charge and discharge durations for both SCs are roughly similar, suggesting high coulombic efficiency and good electrochemical reversibility. Additionally, the discharge time for the adhesive SC is considerably longer than that of the original counterpart, thanks to the “mucus-coating” structure. Furthermore, the GCD curves indicate minimal iRdrop values, highlighting a key characteristic of reversible capacitance and low internal resistance [41].

Additionally, Figure 4c illustrates the specific capacitances recorded at different current densities for both SCs. The adhesive SC exhibits significantly higher specific capacitances across all current densities compared to the original counterpart, suggesting enhanced performance rates. At a current density of 0.30 A/g, the capacitance of the adhesive SC (249.3 F/g, at 0.8 V) is approximately 1.3 times greater than that of the original SC (189.7 F/g, at 0.8 V) and represents about 74.8% of the peak specific capacitance observed at 0.15 A/g, indicating that it retains strong electrochemical performance even at elevated current densities. The cycling stability of both SCs was assessed through GCD testing at 0.5 A/g over 2000 cycles (Figure 4d). The retention of specific capacitance for both types decreased as the number of cycles increased. After completing 20,000 cycles, the adhesive SC preserved 73.4% of its initial capacitance, showcasing its reliable cyclic durability.

To demonstrate its practical application, three adhesive SCs are connected both in series and in parallel, with their performance assessed through GCD measurements, as depicted in Figure 4e. In the series configuration, the voltage range increases to 2.4 V, which is three times that of an individual SC. Additionally, the charge and discharge duration at a constant current density of 0.15 A/g remains largely the same for both the series and single configurations, suggesting that the capacitive capabilities of each SC are effectively maintained in the series setup. In the parallel arrangement, the discharge duration is tripled, indicating that the capacitance stored is also three times greater. This functionality is demonstrated by successfully powering three LEDs for approximately 5 min, as shown in Figure 4f.

### 2.5. Impact of Mechanical Strain on the Electrochemical Characteristics of the XG/HPAAm/NaCl DN HPE SC

**Figure 5:**
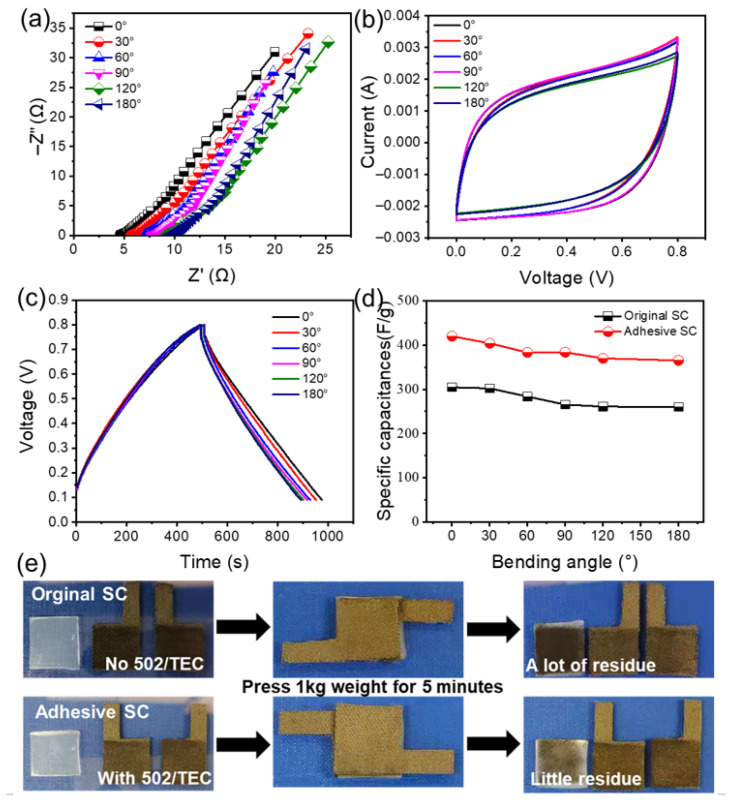
Effect of deformation on the electrochemical characteristics of original and adhesive SCs: (**a**) EIS curves at bending angles ranging from 0 to 180°; (**b**) CV curves recorded at 300 mV/s across various bending angles; (**c**) GCD curves for the adhesive SC at a current density of 1.2 A/g under different bending angles; (**d**) specific capacitance across bending angles from 0 to 180°; (**e**) optical images of components before/after assembly and after 5-min vertical application of 1 kg weight.

To assess the adaptability of the SC, the electrochemical characteristics of original and adhesive SCs are evaluated at bending angles of 0°, 30°, 60°, 90°, 120°, and 180° (Figure 5 and Figure S9). As illustrated in Figure 5a and Figure S9a, the *R*_ct_ values for the original and adhesive SCs rise by 5.6 times and 0.6 times, respectively, as the bending angle progresses from 0° to 180°. This increase is attributed to the reduced bonding between the electrode and the electrolyte [42]. In Figure 5b and Figure S9b, the adhesive SC maintains a CV curve similar in size to that of the initial sample, even at 180°. Conversely, the original SC begins to lose its rectangular shape as the bending angle increases. The GCD curves presented in Figure 5c and Figure S9c reveal that the capacitance values for the adhesive SC at different bending angles are calculated as 420.12 F/g at 0° and 365.25 F/g at 180°, indicating a gradual decline in capacitance with increasing bending angles. Notably, the capacitance loss is only 13.1% at 180° for the adhesive SC, which is less than the 14.6% loss observed in the original SC (Figure 5d), highlighting the enhanced flexibility of the adhesive SC. The specific capacitance, cycling stability, and bending retention values are provided in Table S3.

The ability to endure repeated bending is crucial for the effective use of flexible SCs. The GCD tests were performed at angles of 60° and 90°, with the results for specific capacitance illustrated in Figure S10a,b. In Figure S10a, the original SC exhibits a significant decline in specific capacitance after 5000 cycles of bending at 60° (only 58.6% retention), highlighting its inadequate bending stability. In contrast, the adhesive SC shows a much smaller reduction over the same number of cycles (84.6% retention), indicating its improved stability under bending. Likewise, in the 90° bending test depicted in Figure S10b, the adhesive SC experiences only a minor drop (79.7% retention), further validating its good bending resilience. This slight reduction is linked to insufficient contact between the electrolyte and the electrode during bending [41,43]. Additionally, the *R*_ct_ value progressively rises with the increasing number of bending cycles, as illustrated in Figure S11.

The significant variation in bending stability observed between the two samples is effectively illustrated by the optical images presented in Figure 5e. The original SC retains a considerable amount of residue after being subjected to a 1 kg weight for 5 min, highlighting its inadequate adhesion. This lack of strong adhesion results in considerable contact loss between the electrode and the electrolyte during bending cycles, leading to a notable decline in capacitance. On the contrary, the adhesive SC treated with 502/TEC mucus leaves very little residue after pressing, indicating its robust adhesion. This strong physical interaction enables much better contact between the electrolyte and the electrode, even during repeated bending, which explains the minimal capacitance reduction seen in the samples with adhesive SC.

**Figure 6:**
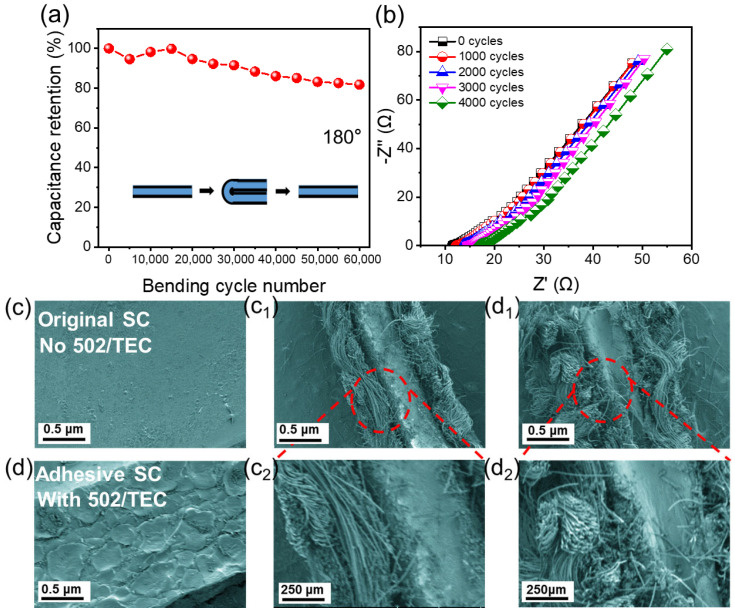
Cycle experimental results of 180° bending and 180°–peeling tests on adhesive SC. (**a**) Capacitance retention during 60,000 bending cycles, (**b**) EIS curves during 4000 bending cycles. SEM images of the surface and cross-section of the original and adhesive HPE-based SCs. (**c**) Surface of the original SC without 502/TEC; (**d**) Surface of the adhesive SC with 502/TEC; (**c_1_**,**c_2_**) Cross-sectional views of the original SC after bending cycles; (**d_1_**,**d_2_**) Cross-sectional views of the adhesive SC after bending cycles.

To assess the cyclic stability during significant deformations, tests involving 180° bending and peeling tests were conducted. The adhesive SC exhibits a minor decline in capacitance retention compared to the original SC, which demonstrates improved stability, as illustrated in Figure S12a. In Figure S12b, it is evident that the original SC experiences interface separation and damage after 4000 bending cycles, while the adhesive SC maintains a stronger bond and greater integrity, highlighting its structural stability advantage, in line with Figure S12a. Notably, even after enduring 60,000 bending cycles, the adhesive SC retains a capacitance of approximately 80% (Figure 6a). The observed reduction in capacitance retention is attributed to water loss from the hydrogel during the electrochemical cycling, which can extend from several hours to days in the air without encapsulation (with humidity < 50%) [39,44]. Figure 6b presents the EIS curves for the adhesive SC after 0–4000 bending cycles. The curves show a good overlap as the number of cycles increases, indicating that the charge-transfer resistance remains relatively stable. Consequently, the material demonstrates consistent electrochemical charge transfer during bending, showcasing good electrochemical stability and mechanical strength for applications in flexible device applications.

The SEM images in Figure 6c,d provide insights into the structural characteristics that underpin the previously mentioned mechanical and electrochemical properties. In Figure 6c, the HPE of the original SC shows a relatively smooth surface at low magnification, while at higher magnifications in Figure 6(c_1_,c_2_), the cross-section of the original SC exhibits a sparse arrangement of carbon fibers with poor interconnectivity. This sparse configuration may result in inadequate binding with the hydrogel electrolyte, making it more prone to detachment from the carbon cloth during bending and cycling, which can elevate interface resistance and diminish electrochemical performance. Conversely, Figure 6d illustrates the HPE of the adhesive SC modified with the 502/TEC “mucus-coating”, exhibiting a distinct surface morphology. According to the SEM images (Figure 6c,d), the 502/TEC “mucus-coating” penetrates into the gaps between carbon fibers and forms a conformal, continuous layer on the electrode surface. This coating improves the physical contact and interfacial compatibility between the hydrogel electrolyte and the carbon cloth electrode, thereby enhancing the interfacial stability. The improved structural integrity observed in the SEM images directly supports the enhanced bending resistance and cycling stability of the adhesive SC. The robust fiber arrangement in the adhesive SC is better equipped to endure repeated bending and cycling, thereby minimizing the likelihood of structural damage. This stable interface between the fibers and electrolyte is crucial for sustaining consistent electrochemical performance, aligning with the observed high capacitance retention and stable EIS results. The improved interfacial adhesion is mainly attributed to the conformal coverage and infiltration of the 502/TEC coating, which enhances the physical interlocking and contact area at the hydrogel–electrode interface. This stabilization effect prevents interfacial delamination during repeated bending, which is consistent with the observed high capacitance retention under large deformation.

**Figure 7:**
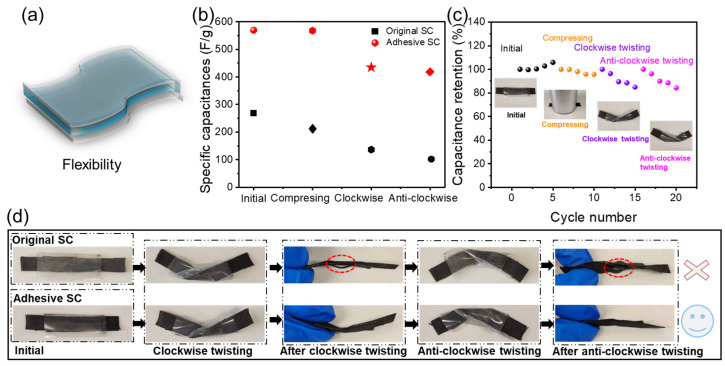
The complex deformation of the original and adhesive HPE SCs. (**a**) Deformation schematic diagram of flexible SC, (**b**) specific capacitance, (**c**) capacitance retention rate after 5 cycles, and (**d**) optical photograph.

Additionally, we examined the electrochemical characteristics of original and adhesive SCs under various deformation scenarios, the deformation schematic diagram as illustrated in Figure 7a. In Figure 7b, the specific capacitances of both types of SCs are displayed for their initial and deformed states (including compression and both clockwise and anti-clockwise twisting). The findings indicate that the adhesive SC consistently demonstrates higher specific capacitance under these conditions when compared to the original SC. Figure 7c reveals that the initial capacitance retention is high in the initial state, but it diminishes as the number of cycles increases. Notably, the capacitance retention during compression remains relatively stable, unlike the twisting deformations. This stability can be attributed to the reliable shape recovery properties of the DN gel electrolyte [29] and the bonding quality between the electrolyte and the electrode during the deformation. Consequently, we performed 5000 clockwise twisting tests on both SC types, with the optical images presented in Figure 7d. The images indicate that the interface between the electrolyte and the electrode interface in the original SC shows significant layering, whereas the interface in the adhesive SC is well bonded. This observation correlates with the electrochemical performance variations depicted in Figure 7b,c. In summary, the adhesive SC improved the interactions at the electrode–electrolyte interface, leading to enhanced electrochemical performance and resistance to mechanical deformation.

## 3. Conclusions

The excellent performance of our flexible supercapacitors arises from the combination of two key innovations: the robust DN hydrogel electrolyte provides a stable substrate, while the 502/TEC mucus-coating ensures reliable electrode–electrolyte contact under repeated deformation. We introduced a strategy inspired by the mucus of a snail, which combines a robust DN HPE with a 502/TEC coating surface resembling a “mucus” to create flexible SCs. This method effectively improves both the mechanical durability and electrochemical efficiency of the devices. The resulting tough HPE demonstrates high stretchability, toughness, and ionic conductivity, allowing the flexible devices to endure various mechanical stresses. Additionally, the “mucus-coating” electrode/electrolyte interface represents a key advancement: it ensures strong adhesion of electrode materials, enhances interfacial charge transfer and capacitance, and guarantees consistent performance retention under different deformations. For example, when bent at 180°, the device’s lifespan can increase from 5000 cycles to 60,000 cycles, while retaining over 80% of its capacitance. Importantly, this approach is adaptable and paves the way for the development of innovative functional flexible devices. For instance, tough HPEs with added features (such as anti-freezing or shape memory capabilities) could be investigated for use in stretchable electronics [45,46,47]. Furthermore, microstructured surfaces can be strategically designed using photolithography to create 3D interfaces with adjustable adhesion, surface area, or optical characteristics [48,49]. Future work will focus on further improving the long-term practical stability of the device, including evaluating storage stability, dehydration resistance, and electrochemical performance under prolonged exposure to ambient conditions, which are critical for real-world flexible device applications.

## 4. Materials and Methods

### 4.1. Materials

Acrylamide (AM, AR, 99.0%), xanthan gum (XG), triethyl citrate (TEC), stearyl methacrylate (SMA), and sodium dodecyl sulfate (SDS) were sourced from Shanghai Aladdin Bio-Chem Technology Co., Ltd. (Shanghai, China). The photoinitiator 1-hydroxy cyclohexyl phenyl ketone (Irgacure-184, 98%) was kindly provided by Bide Pharmatech Co., Ltd. (Shanghai, China). Sodium chloride (NaCl) and α-Cyanoacrylate ethyl ester (502 glue) were purchased from Fuchen Chemical Regent Factory (Tianjin, China). Activated carbon (AC) was obtained from Shanghai Sino Tech Investment Management Co., Ltd. (Shanghai, China), while carbon cloth (CC) was sourced from Shanghai Hesen Electric Co., Ltd. (Shanghai, China). All chemicals were utilized as received without any additional purification.

### 4.2. Preparation of XG/HPAAm/NaCl DN HPEs

The DN HPE composed of XG/HPAAm/NaCl was created using a one-step synthesis approach. In this process, 2 g of AM monomer, a specific quantity of XG (with varying concentrations of 0, 1, 2, 3, and 4 wt%), N,N′-methylenebisacrylamide (MBA, 0.2 mol% relative to AM monomer) as cross-linking agent, 1-hydroxy cyclohexyl phenyl ketone (Irgacure-184, 1.0 wt% relative to the monomer solution) as UV initiator, and other necessary components were introduced into a 7 wt% SDS solution containing 0.5 M NaCl. The synthesis involved a heating and photopolymerization technique. Initially, all components were mixed into the SDS/NaCl aqueous solution, which was then heated and stirred at 95 °C for one hour to achieve a uniform mixture. Following this, nitrogen gas was introduced to eliminate oxygen from the solution for 30 min. The resulting mixture was then placed between two glass plates, separated by a silicone rubber spacer of 1.8 mm thick, and into plastic molds measuring 10 mm in diameter and 10 ± 1 mm in height. It was subsequently exposed to UV light (λ = 365 nm, irradiation intensity: 10 mW/cm^2^) for 2 h under nitrogen atmosphere to produce the desired hydrogel electrolyte membranes. During the heating phase, XG generates linear macromolecules, forming the first network, while SMA integrates into SDS micelles, creating polymerizable micelles. The copolymerization of SMA and AM leads to the interconnection of various polymer chains, resulting in the formation of the HPAAm network, which serves as the second network. Additionally, hydrogen bonding occurs between the HPAAm and XG networks.

### 4.3. Preparation of the AC Electrode and Assembly of Flexible SCs

The HPE was sliced into squares measuring roughly 2 × 2 cm^2^ and had a thickness of about 1 mm. A brush was used to carefully apply 502 glue (200 μL/cm^2^)—a widely recognized cyanoacrylate adhesive renowned for its rapid curing and high adhesion efficiency—onto both sides of the HPE, forming mucus-mimetic coatings. To enhance the flexibility and extensibility of the adhesive while retaining a certain degree of elasticity post-curing, 20 vol% TEC was incorporated into the 502 glue before application. Notably, the incorporation of 20 vol% TEC prevents the adhesive from forming a fully dense insulating layer during curing. Instead, it forms a thin, uniform adhesive layer at the interface that provides strong interfacial bonding without blocking the inherent ion-transport pathways between the HPE and the AC electrode. This mixed adhesive coating is a separate chemical agent and does not involve any modification of the XG/HPAAm hydrogel network itself. After this, electrodes made of CC/AC were affixed to either side, resulting in the assembly of a flexible SC (Figure S13b). A mixture of AC (80 wt%), acetylene black (10 wt%), and a PTFE aqueous solution binder (10 wt%) was blended in ethanol at room temperature to create a consistent slurry. This slurry was then evenly applied to a CC substrate that had been pre-treated and subsequently dried at 60 °C for 12 h. The SC was constructed by placing the HPE between two electrodes made of AC, with the amount of AC on each electrode carefully regulated to 0.1 mg/cm^2^. To prevent moisture from entering and to reduce experimental variability, the assembled SC was carefully wrapped in a polyethene film before testing its performance.

To facilitate differentiation, the SC featuring “mucus-coating” formations on the surface of the HPE was labeled as “Adhesive SC”, while the control group was labeled as “Original SC”.

### 4.4. Characterizations

The surface structure of HPEs was examined using a scanning electron microscope (VEGA3-XMU, TESCAN, Brno, Czech Republic). A WDW-GD universal testing machine (Shanghai Songton Instrument Co., Ltd., Shanghai, China) was employed for mechanical assessments of the HPE. A dumbbell-shaped specimen underwent tensile testing at a speed of 100 mm/min. The HPEs were sliced into two sections with a blade, which were then brought into contact at varying temperatures for a specified duration. The mechanical measurements were used to assess the self-healing capabilities of the HPE. To determine the bonding strength at the interface between the electrolyte and the electrode, rectangular SC samples (6 cm long, 2 cm wide) containing HPEs—some treated with a “mucus” method and others without—were subjected to a continuous 180° peeling test at a strain distance of 4 mm and a peeling speed of 50 mm/min, with no breaks during the procedure. A minimum of three samples were tested for each experimental condition to ensure accurate results.

### 4.5. Electrochemical Measurements

The resistance measurement of the XG/HPAAm/NaCl DN HPEs membrane was performed using EIS with a CHI760E electrochemical workstation from Shanghai Chenhua Co., Ltd., Shanghai, China. The tests were conducted over a frequency range of 0.1 Hz to 100 kHz, applying an amplitude of 5 mV at ambient temperature. Circular samples (radius = 0.75 cm) were extracted from the XG/HPAAm/NaCl DN HPE and placed between two stainless steel (SS) blocking electrodes to create a cell, which was then enclosed in a PTFE mold (see Figure S1a). Nyquist plots were generated for each electrolyte, and the resistances were determined from their intercepts on the *x*-axis. The ionic conductivity σ (S/cm) was calculated using the following formula:

(1) $$\sigma =\frac{L}{A\times R_{b}}$$

where *A* denotes the interface area (cm^2^) between the HPE and the current collectors, L signifies the thickness (cm) of the HPE, and *R_b_* refers to the bulk resistance (Ω) measured through impedance analysis. The electrochemical stability range was assessed via CV using the same electrochemical setup, with a potential range of −2 V to 2 V at a scanning speed of 10 mV/s.

The EIS of the SC was carried out across a frequency spectrum of 0.1 Hz to 100 kHz. GCD cycling was evaluated using a battery testing device (CT 2001A, LAND Technology Co., Ltd., Wuhan, China) at varying current densities within a voltage range of 0.0–0.8 V. The CV analysis of the SC was executed at different scan rates, ranging from 1 to 300 mV/s, also within the 0.0–0.8 V potential range. The specific capacitance of the SC (*C*, F/g) and the specific capacitance of the electrode (*C_s_*, F/g), along with the energy density (*E*, Wh/kg) and power density (*P*, W/kg) of the SC, can be calculated from the GCD curves using the following formulas [19,50,51]:

(2) $$C=\frac{I\times \Delta t}{\Delta V\times m}$$

(3) $$C_{S}=4\times C$$

(4) $$E=\frac{1}{8}\times C_{S}\times {\Delta V}^{2}\times \frac{1000}{3600}$$

(5) $$P=\frac{E}{\Delta t}\times 3600$$

where I represents the current during discharge, ∆*t* denotes the duration of the discharge, and ∆*V* indicates the change in voltage that occurs after the internal resistance drop (iRdrop). Additionally, m refers to the mass of the active materials (AC) present on both electrodes.

All electrochemical measurements of the supercapacitor were prepared using a two-electrode system.

## Data Availability

The original contributions presented in this study are included in the article. Further inquiries can be directed to the corresponding author.

## Supplementary Materials

The following supporting information can be downloaded at: https://www.mdpi.com/article/10.3390/gels12050441/s1, Table S1. Mechanical and ionic conductivity properties of XG/HPAAm/0.4 M NaCl DN HPE with different XG concentrations. Figure S1. CV curves of XG/HPAAm/0.4 M NaCl DN HPE with various XG contents. Figure S2. Effect of NaCl levels on the (a) tensile stress-strain curves and (b) tensile strengths and elongations of the 2 wt% XG-HPAAm/NaCl DN HPE membrane. (c) Exhibits significant flexibility, capable of stretching, twisting, and withstanding knotting of the 2 wt% XG-HPAAm/NaCl DN HPE membrane (0.4 M NaCl). Figure S3. Effect of NaCl levels on the (a) EIS curves, and (b) ionic conductivity of the 2 wt% XG-HPAAm/NaCl DN HPE membrane. Table S2. Effects of NaCl concentration on mechanical and ionic conductivity properties of 2 wt% XG/HPAAm/NaCl DN HPE. Figure S4. CV curves of 2 wt% XG/HPAAm/NaCl DN HPE with various NaCl contents. Figure S5. Mechanical characteristics and ionic conductivity of the 2 wt% XG/HPAAm/0.4 M NaCl DN HPE post-healing. (a) The tensile stress-strain before and following a 1-hour healing period at various temperatures; (b) ionic conductivity measured before and after a 10-minute healing at 70 °C, after 10 cycles of cutting and healing. Figure S6. Mechanical properties of the XG/HPAAm/NaCl DN HPE after healing. (a) The tensile strengths and elongation values, and (b) healing efficiency before and after healing for 1 h at different temperatures; (c) tensile strengths and elongation values before and after healing for 70 °C at different healing times. Figure S7. Mechanical characteristics and ionic conductivity of the 2 wt% XG/HPAAm/0.4 M NaCl DN HPE post-healing. (a) The tensile stress-strain curves, and (b) healing efficiency before and after the healing process at 70 °C across different durations. (c) Proposed healing mechanism. Figure S8. Electrochemical performance of original and adhesive SCs. (a) EIS curves, (b) CV curves of the original SC at various scan rates from 1 to 300 mV/s, and (c) GCD curves of the original SC at various charging/discharging current densities from 0.15 to 1.2 A/g. Figure S9. Influence of deformation on the electrochemical properties of the original SC. (a) EIS curves at bending angle from 0 to 180°, (b) CV curves at different bending angles at 300 mV/s, and (c) GCD curves at various bending angles at a current density of 1.2 A/g. Table S3. Electrochemical performance of original and adhesive supercapacitors. Figure S10. Specific capacitance alterations observed during (a) 20,000 cycles at a 60° bend and (b) 5,000 cycles at a 90° bend for original and adhesive SCs. Figure S11. Cyclic EIS curves of (a) original and (b) adhesive SCs at a bending angle of 60 °. Figure S12. (a) Variation of specific capacitance during 4000 bending cycles (bending angle = 180°) of the original and adhesive SCs, (b) optical photograph of the interface between the electrode and the electrolyte of the capacitors after 4000 bending cycles. Figure S13. (a) Illustration of the mold used for electrochemical testing of HPE, (b) illustration of the flexible SC design.
