# Supplementary material for: Snail Mucus-Inspired Interface: A Resilient and Self-Healing Double-Network Hydrogel Polymer Electrolyte for Flexible Supercapacitors
Source: Gels. 2026 May 17;12(5):441. doi: 10.3390/gels12050441 (PMC13205218; doi:10.3390/gels12050441)
Supplement: Supplementary file 1 [file gels-12-00441-s001.zip › gels-4280531-supplementary.pdf]

gels

Article

Snail Mucus-Inspired Interface: A Resilient and Self-Healing Double-Network Hydrogel Polymer Electrolyte for Flexible Supercapacitors

Mengxiao Wang [1], Jia Yang [2], Gang Qin [2*] and Qiang Chen [3*]

[1] School of Electrical Engineering, Zhejiang University of Water Resources and Electric Power, Hangzhou 310018, China
[2] School of Materials Science and Engineering, Henan Polytechnic University, Jiaozuo 454000, China
[3] Wenzhou Institute, University of Chinese Academy of Sciences, Wenzhou 352001, China
* Correspondence: qingang@hpu.edu.cn (G. Q.); chenqiang@ucas.ac.cn (Q. C.)

Table S1. Mechanical and ionic conductivity properties of XG/HPAAm/0.4 M NaCl DN HPE with different XG concentrations.

XG concentration (wt%)	Fracture stress (MPa)	Elongation at break (mm/mm)	Ionic conductivity (S/m)
0	0.226±0.070	9.398±1.503	4.337±0.359
1	0.358±0.067	10.38±0.916	3.607±0.245
2	0.367±0.063	9.502±0.721	3.580±0.593
3	0.438±0.065	8.995±0.867	3.337±0.025
4	0.494±0.050	8.690±0.659	3.007±0.190

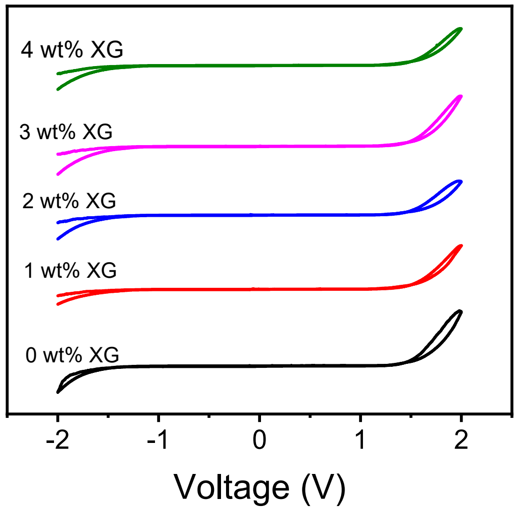

Figure S1. CV curves of XG/HPAAm/0.4 M NaCl DN HPE with various XG contents.

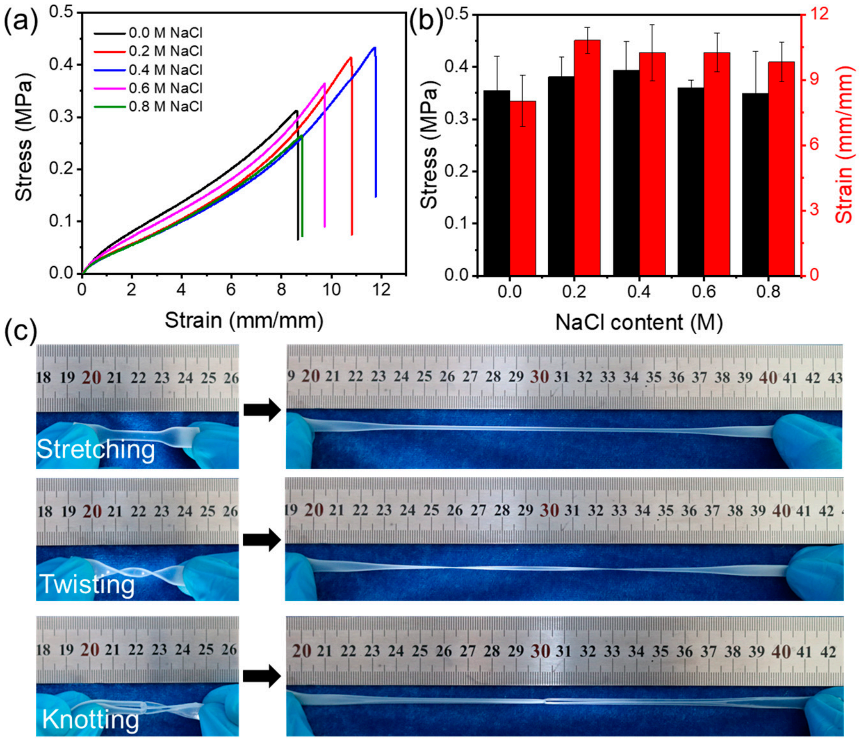

Figure S2. Effect of NaCl levels on the (**a**) tensile stress-strain curves and (**b**) tensile strengths and elongations of the 2 wt% XG-HPAAm/NaCl DN HPE membrane. (**c**) Exhibits significant flexibility, capable of stretching, twisting, and withstanding knotting of the 2 wt% XG-HPAAm/NaCl DN HPE membrane (0.4 M NaCl).

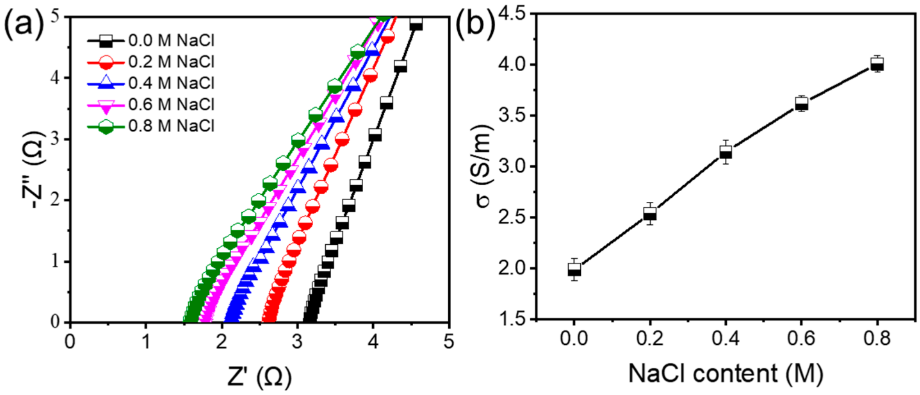

Figure S3. Effect of NaCl levels on the (**a**) EIS curves, and (**b**) ionic conductivity of the 2 wt% XG-HPAAm/NaCl DN HPE membrane.

Table S2. Effects of NaCl concentration on mechanical and ionic conductivity properties of 2 wt% XG/HPAAm/NaCl DN HPE.

NaCl concentration (M)	Fracture stress (MPa)	Elongation at break (mm/mm)	Ionic conductivity (S/m)
0.0	0.312±0.065	8.616±1.174	1.987±0.110
0.2	0.411±0.085	10.741±1.105	2.537±0.110
0.4	0.432±0.055	11.736±1.256	3.580±0.119
0.6	0.364±0.015	9.735±0.918	3.617±0.075
0.8	0.263±0.080	8.820±0.911	4.007±0.081

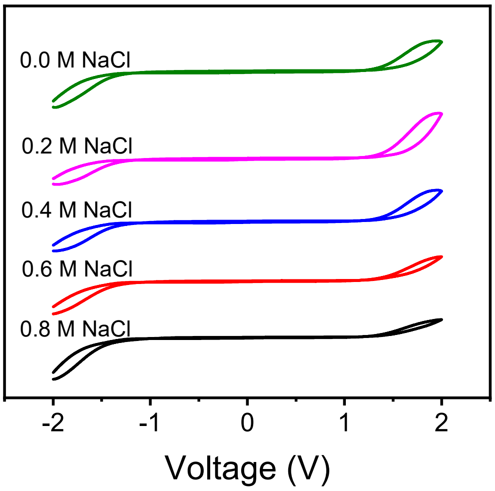

Figure S4. CV curves of 2 wt% XG/HPAAm/NaCl DN HPE with various NaCl contents.

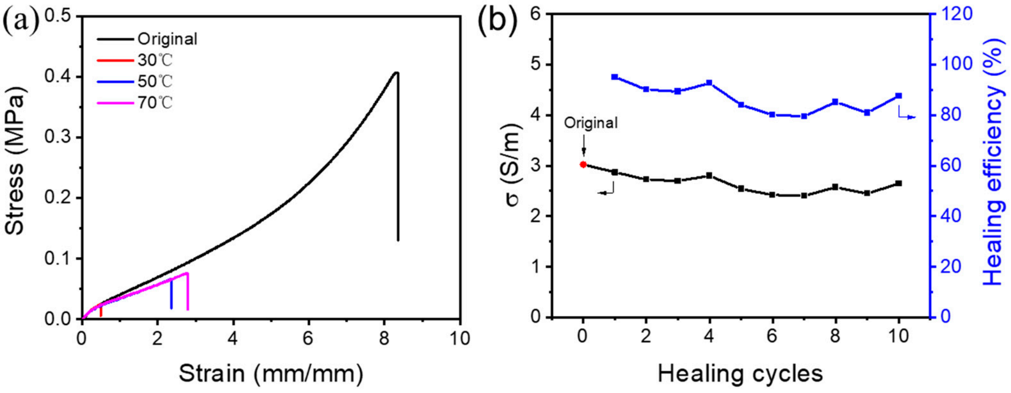

Figure S5. Mechanical characteristics and ionic conductivity of the 2 wt% XG/HPAAm/ 0.4 M NaCl DN HPE post-healing. (**a**) The tensile stress-strain before and following a 1-hour healing period at various temperatures; (**b**) ionic conductivity measured before and after a 10-minute healing at 70 °C, after 10 cycles of cutting and healing.

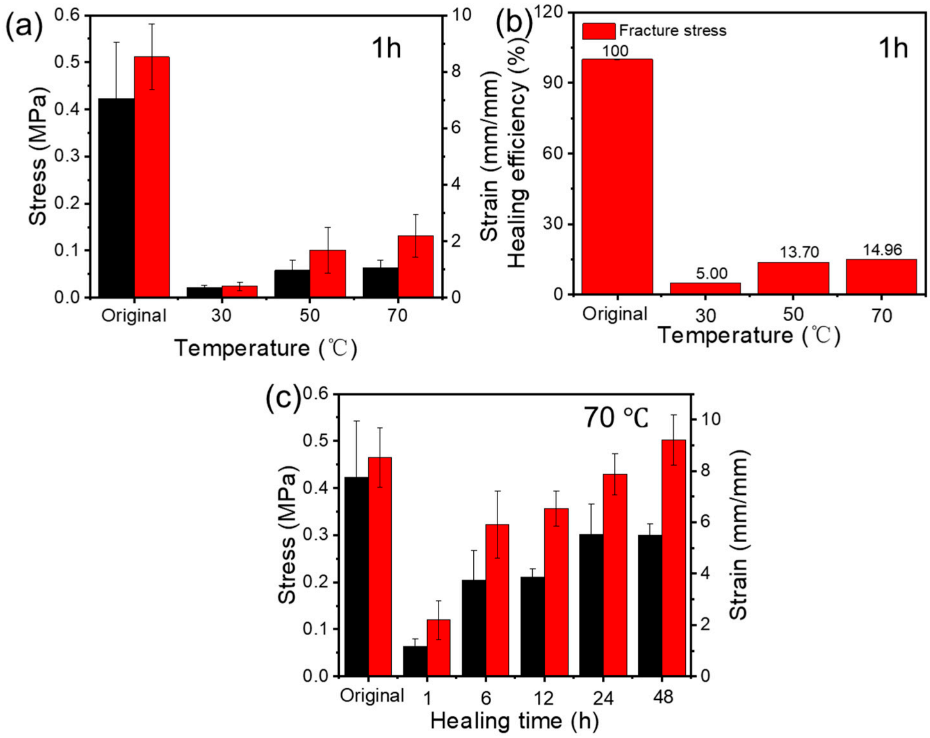

Figure S6. Mechanical properties of the XG/HPAAm/NaCl DN HPE after healing. (**a**) The tensile strengths and elongation values, and (**b**) healing efficiency before and after healing for 1 h at different temperatures; (**c**) tensile strengths and elongation values before and after healing for 70 °C at different healing times.

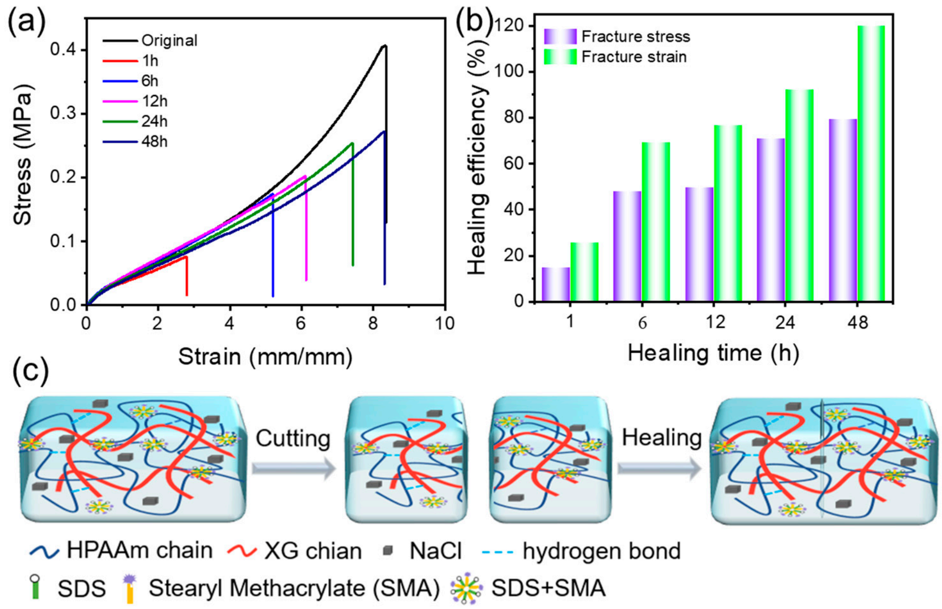

Figure S7. Mechanical characteristics and ionic conductivity of the 2 wt% XG/HPAAm/0.4 M NaCl DN HPE post-healing. (**a**) The tensile stress-strain curves, and (**b**) healing efficiency before and after the healing process at 70 °C across different durations. (**c**) Proposed healing mechanism.

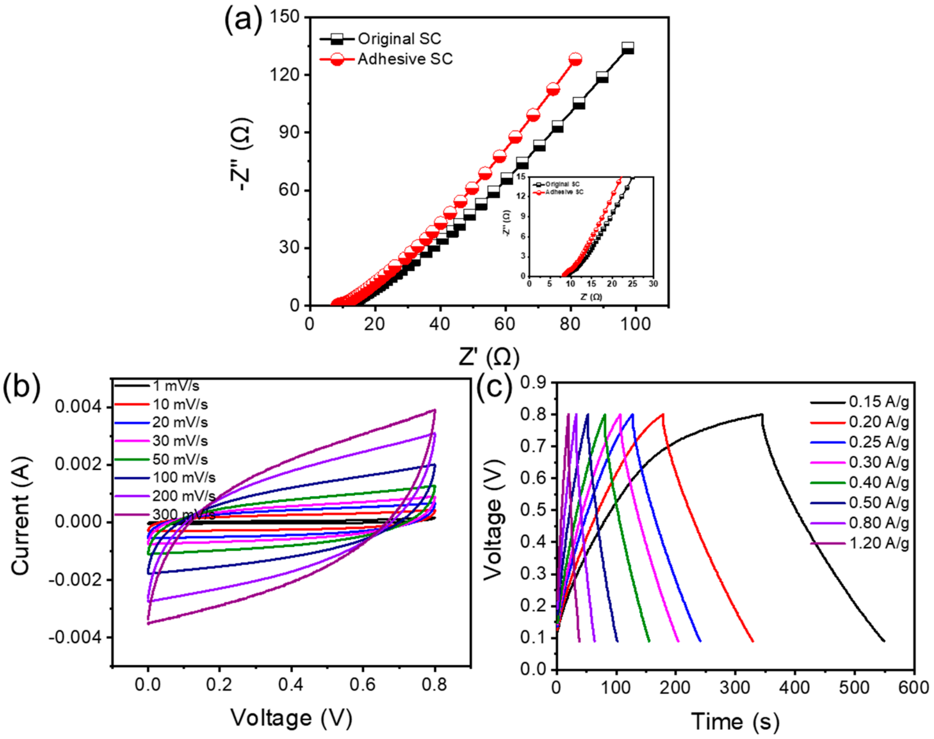

Figure S8. Electrochemical performance of original and adhesive SCs. (**a**) EIS curves, (**b**) CV curves of the original SC at various scan rates from 1 to 300 mV/s, and (**c**) GCD curves of the original SC at various charging/discharging current densities from 0.15 to 1.2 A/g.

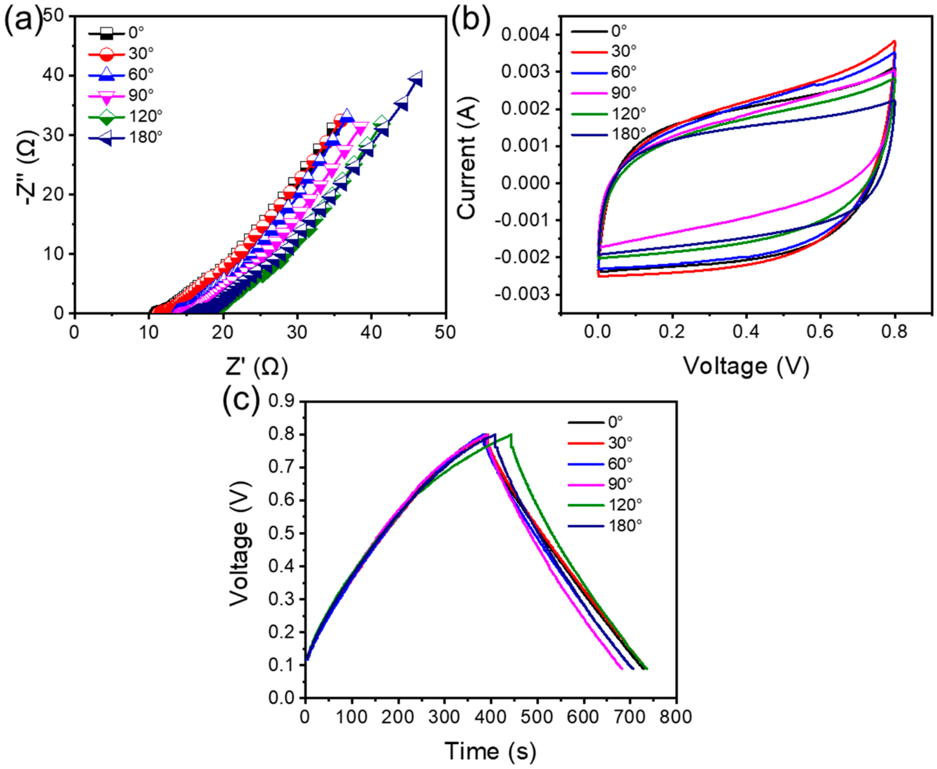

Figure S9. Influence of deformation on the electrochemical properties of the original SC. (**a**) EIS curves at bending angle from 0 to 180°, (**b**) CV curves at different bending angles at 300 mV/s, and (**c**) GCD curves at various bending angles at a current density of 1.2 A/g.

Table S3. Electrochemical performance of original and adhesive supercapacitors.

Sample	R_{ct} (Ω)		Specific capacitance (F/g, 1.2 A/g)		Retention after 20000 cycles
	0°	Bending at 180°	0°	Bending at 180°	
Original SC	0.560	3.696	304.663	260.131	40.5
Adhesive SC	0.461	0.738	420.117	365.249	73.4

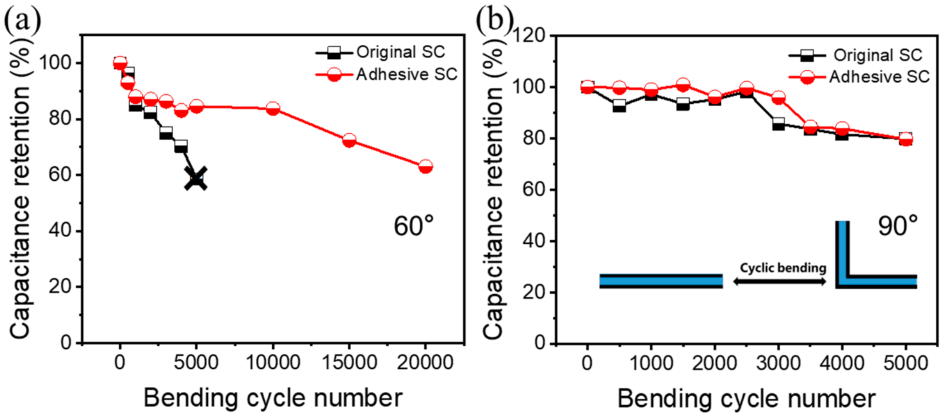

Figure S10. Specific capacitance alterations observed during (**a**) 20,000 cycles at a 60° bend and (**b**) 5,000 cycles at a 90° bend for original and adhesive SCs.

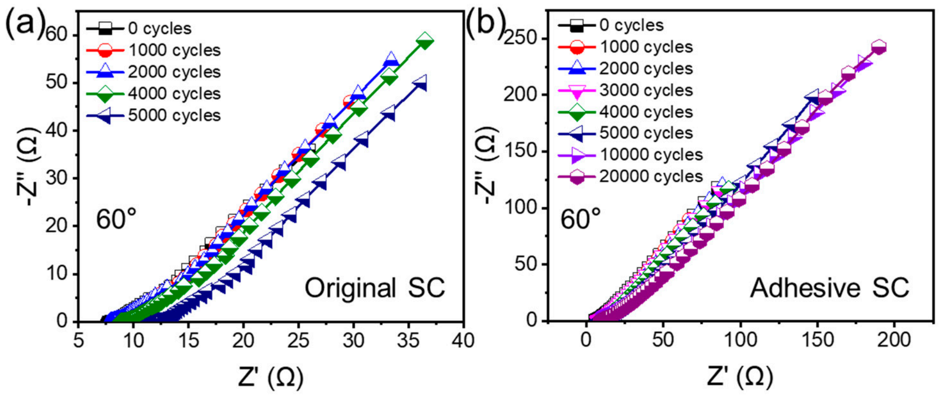

Figure S11. Cyclic EIS curves of (**a**) original and (**b**) adhesive SCs at a bending angle of 60 °.

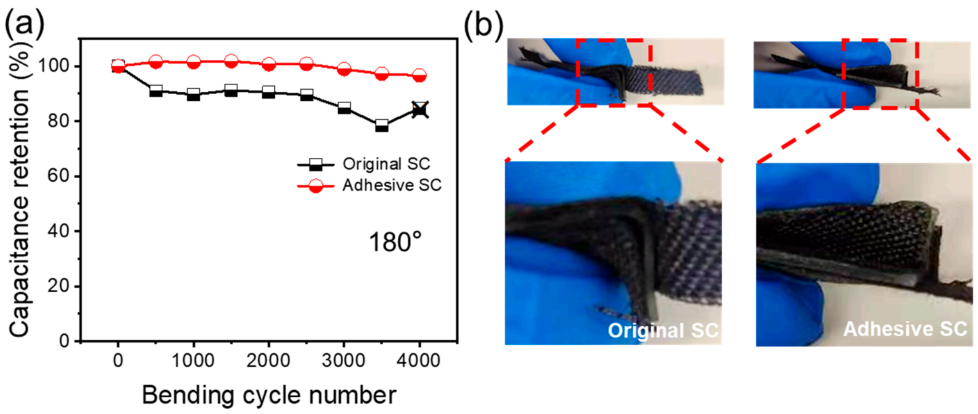

Figure S12. (**a**) Variation of specific capacitance during 4000 bending cycles (bending angle = 180°) of the original and adhesive SCs, (**b**) optical photograph of the interface between the electrode and the electrolyte of the capacitors after 4000 bending cycles.

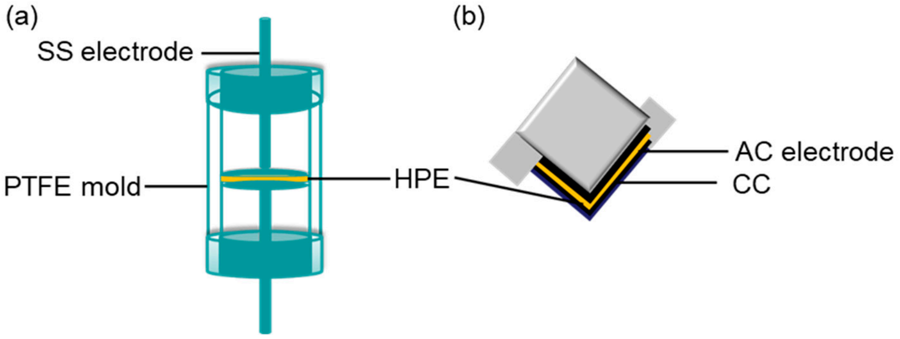

Figure S13. (**a**) Illustration of the mold used for electrochemical testing of HPE, (**b**) illustration of the flexible SC design.

Tentative submission date: April 10, 2026
